# Effects of fascial manipulation, vibration exercise, motor imagery, or neuro‐muscular electrical stimulation on the coagulation system: A pilot study

**DOI:** 10.14814/phy2.70165

**Published:** 2025-01-02

**Authors:** Gerhard Cvirn, Anna Hawliczek, Axel Schlagenhauf, Bianca Brix, Karin Schmid Zalaudek, Sebastian Schwaminger, Margret Paar, Willibald Wonisch, Thomas Wagner, Ziva Arko, Nandu Goswami

**Affiliations:** ^1^ Division of Medicinal Chemistry, Otto Loewi Research Center Medical University of Graz Graz Austria; ^2^ Gravitational Physiology and Medicine Research Unit, Division of Physiology, Otto Loewi Research Center Medical University of Graz Graz Austria; ^3^ Division of General Paediatrics, Department of Paediatrics and Adolescent Medicine Medical University of Graz Graz Austria; ^4^ Department of Blood Group Serology and Transfusion Medicine Medical University of Graz Graz Austria; ^5^ Department of Physiotherapy Univerza Alma Mater Europaea Maribor Slovenia; ^6^ Center for Space and Aviation Health Mohammed Bin Rashid University of Medicine and Health Sciences Dubai UAE

**Keywords:** clot formation process, hemodilution, rehabilitation treatment, thrombelastometry, thrombin generation

## Abstract

Available evidence suggests that various medical/rehabilitation treatments evoke multiple effects on blood hemostasis. It was therefore the aim of our study to examine whether fascial manipulation, vibration exercise, motor imagery, or neuro‐muscular electrical stimulation can activate the coagulation system, and, thereby, expose patients to thrombotic risk. Ten healthy young subject were enrolled in the study. Blood samples were obtained pre and posttreatment. Besides standard laboratory methods, calibrated automated thrombography (CAT) and thrombelastometry (TEM) were used allowing sensitive detection of hyper‐ and hypocoagulable states. Application of fascial manipulation, motor imagery, or neuro‐muscular electrical stimulation had vitually no effect whereas a single bout of vibration exercise caused significant coagulation activation. For example, TEM‐derived coagulation times were significantly shortened (209 ± 34 vs. 187 ± 41 s, *p* = 0.0098) and CAT‐derived thrombin peaks were significantly higher (235 ± 88 vs. 268 ± 82 nM, *p* = 0.0020) in post compared with preexercise samples. Moreover, vibration exercise, motor imagery, and neuro‐muscular electrical stimulation caused significant plasma expansion (6.15%, 7.53%, and 3.88% plasma volume changes, respectively). We conclude that vibrational exercise apparently represents a potential procoagulant stimulus, and ongoing studies have to clarify whether VE should be applied particularly to patients with an elevated risk for thrombosis.

## 1 INTRODUCTION

Various treatments are currently used in medical/sports rehabilitation, space medicine, or in geriatrics in order to restore health or mitigate the progression of a disease, aging (Goswami et al., 2017) or spaceflight induced decondtioning (Ahmed et al., 2024; Goswami, 2017; Zuccarelli et al., 2024). The present study deals with four of these interventions: (i) fascial manipulation (FM), (ii) vibration exercise (VE), (iii) motor imagery (MI), and (iv) neuro‐muscular electrical stimulation (NMES). We examined the effects of these interventions on the haemostatic system in ten healthy young participants. Possible intervention‐induced coagulation activation might be an adverse effect particularly for individuals with an elevated risk of thrombosis, for example, for patients with histories of ischemic stroke or cardiovascular diseases, patients with atherosclerosis, (Cvirn et al., 2017) or in astronauts (Elias et al., 2024; Harris et al., 2023; Harris et al., 2022a, 2022b)

Fascia, the soft skeleton of the human body, is a connective tissue which is formed of multiple layers that are gliding (in healthy persons) smoothly among each other to allow proper muscle activation and force transmission. The gliding is enabled by hyaluronic acid (HA). Mechanical factors (surgery, scars), chemical factors (inflammation) or thermic factors (exposure to cold) can cause agglomeration of HA, leading to densifications/restrictions not allowing ideal gliding among the fascial layers. FM aims at resolving the densifications to restore normal function. In the present study FM was applied to eight standard sites on the participants according to Stecco, each site was treated for 2 min (Stecco & Day, 2010). To our knowledge, the influence of FM on the coagulation system has not been investigated so far.

Vibration was provided to the sole of each participant's feet by using the Galileo device. This platform works like a seesaw due to its lateral alternating motion, stimulating a movement pattern similar to human gait. The fast rocking movement of the training platform causes a tilting movement of the pelvis exactly as when walking, but much more often. This device was sucessfully applied to patients with back pain to increase the muscle strenght, it further alters leg blood flow, muscle oxygenation and blood volume (Bruyere et al., 2005; Kawanabe et al., 2007). Studies have shown its safe and positive effecte in chronic obstructive pulmonary disease (COPD), dialysis, multiple sclerosis, lung and heart transplant patients and also in toddlers (Kerschan‐Schindl et al., 2001; Lythgo et al., 2009; Mason et al., 2012; Perchthaler et al., 2015; Rittweger et al., 2002; Seefried et al., 2017; Stark et al., 2016). In the present study, the participants stood on the galileo device for 15 min of vibration at 13 Hz and 2 mm amplitude. To our knowledge, data on the effects of VE particularly on the clot formation process are not available to date.

MI is a mental process in which an individual simulates in his/her mind a movement or motor task without producing an overt action (Jeannerod, 2001). The vegetative and the motor nervous systems are co‐activated during anticipation of the action. In the present study the imagined movement was standing up. MI is applied, for example, for gait rehabilitation after stroke (Silva et al., 2020). To our knowledge, no data concerning the effects of MI on the haemostatic system are available so far.

Neuro‐muscular electrical stimulation (NMES) involves applying an intermittent electrical current to peripheral muscles using special electrodes placed on the skin over the targeted muscles. Electrical current depolarizes motor end‐plates via the motor nerve and induces involuntary muscle contractions which activate a sequence of metabolic and vascular processes very similar to those which accompany normal muscle activity (Pette & Vrbova, 1999). NMES has been shown to improve muscle strength, increase range of motion, reduce edema, decrease atrophy and pain in various chronic diseases (Bourjeily‐Habr et al., 2002; Dobsak et al., 2006; Dubinsky & Miyasaki, 2010; Nuhr et al., 2004). To our knowledge, no data concerning the effects of NMES on the haemostatic system are available to date.

Our study's objective was to determine whether the coagulation system is activated by fascial manipulation, vibration exercise, motor imagery, or neuro‐muscular electrical stimulation, leading to an enhanced risk of thrombosis in patients.

## 2 MATERIALS AND METHODS

The study was carried out in accordance with the WMA Declaration of Helsinki. An approval from the Ethics Committee of the Medical University of Graz was obtained before starting the study (EK 30‐268 ex 17/18). Participants provided written and informed consent.

### 2.1 Subjects

Ten participants (4 women, 6 men) not using drugs known to affect the coagulation system were enrolled in this study. They were nonsmokers with no personal or family history of hypercoagulability or bleeding. The participants were requested to refrain from caffeine intake for 24 h before their appointments, as well as to avoid extreme physical activity for approximately 72 h before the study. The protocol was carried out between 8 am and 1 pm in a cool, quiet, and a partially darkened laboratory. Throughout the study, the room temperature was maintained at 23–24°C and humidity around at 55%–60%. The participants were asked to be barefoot throughout the procedure and wear light, comfortable clothing, which allowed for free movement. The participants characteristics were as follows: 23.3 ± 2.8 years (age), 73.9 ± 16.3 kg (weight), 1,77 ± 0.10 m (height), and 23.4 ± 2.9 kg/m^2^ BMI. A collective of 37 subject was initially enrolled in the study. A subset of 10 participants was singled out. These participants underwent all four treatments and, additionally, passed the screening test which assessed the ability to perform kinaesthetic and visual mental imagery. Each treatment was carried out on a separate day, with an interval of at least 3 days between treatments. The order of treatment was fascial manipulation, vibration exercise, motor imagery, or neuro‐muscular electrical stimulation. The selection criteria for the study included: healthy young females and males between the ages of 18 and 35 years. Each female was asked about their menstrual cycle status and whether they were on medication regulating it in any way. Every appointment was scheduled to fit in the same phase of their cycle. Two phases were identified: the early follicular phase (3–11 days after the start of the period) and the mid‐luteal phase (21–27 days after the beginning of the period). The exclusion criteria were smoking, alcoholism, pregnancy, psychological conditions, heart diseases, history of thrombosis or syncope, medications such as beta‐blockers. In addition, the participants should have had no previous experience with vibration platforms. Once recruited, however, the study participants carried out a familiarization session with the vibration platform.

### 2.2 Treatments

Fascial manipulation: FM was performed according to the Stecco® method (Stecco & Day, 2010). The participants were treated at eight points which are typically involved in lymph flow restrictions in clinical/pathological situations. The pressure applied by the knuckle or elbow of the therapist is between 1, 5 and 2 kg. Each participant was treated for approximately 16 min.

#### 2.2.1 Vibration

Galileo is a training and/or therapy device used for the movement of the legs using resistant vibration. The quality of Galileo medical and training products is ensured by a TÜV supervised quality management system for medical devices according to ISO 13485. The device can be used to train power, strength, elasticity, speed and balance (https://www.galileo‐training.com/files/gis‐0001‐en.jpg). The principle of Galileo stand devices is based on the natural movement of people walking. The vibrations generated by the Galileo training platform can be easily varied in amplitude and frequency regardless of body weight (https://www.galileo‐training.com/at‐deutsch/produkte/galileo‐therapiegeraete/vibrationstherapie.html).

#### 2.2.2 MI

In order to ensure that the sample is not including people with extremely high or low imagery skills, the participants underwent a screening to measure the individual ability to perform kinaesthetic and visual mental imagery. For this purpose the revised version of the Movement Imagery Questionnaire (MIQ‐R) was used (Gregg et al., 2010). Additionally, the absence of muscle activity was controlled with electromyography (EMG) and superficial electrodes. The motor task (standing up) was first demonstrated and then tried by the participants. After that they were requested to stay in a laying position and to mentally simulate the same task at a natural, self‐paced speed and with their eyes open. It was specified that the participants felt themselves performing the task (kinesthetic imagery) rather than just visualizing themselves (visual imagery). A vocal “start” and “stop” signal was given by the investigator. In order to avoid mental fatigue the MI training was performed in blocks: 60 s MI alternated by 30 s rest for 16 min, which will result in a total of about 10 min MI training time.

#### 2.2.3 NMES

Self‐adhesive electrodes (80 × 130 mm; PALS® Platinum, Axelgaard Manufacturing Co., Denmark) were placed on the participants thighs approx. 5 cm below the inguinal fold, and approx. 5 cm above upper patellar border. NMES protocol was set as follows: intermittent simultaneous stimulation by biphasic current, pulse width 400 μs, 2 s ramp‐up time, 8 s period of contraction, 1 s fall‐down time, and 12 s period of relaxation, frequency modulation 10–25 Hz, and maximal intensity 60 mA. Working mode was “on–off” (contraction 8 s, relaxation 12 s). Time of intervention (stimulation period) was 15 min. In the present study we applied TENS (transcutaneous electrical neuro‐stimulation) because it is usually well tolerated by most patients (Maffiuletti et al., 2011).

### 2.3 Blood sampling

Blood samples were collected before and immediately after each treatment. Thrombelastometry measurements were performed in whole blood (WB) samples, thrombin generation measurements (calibrated automated thrombogram and prothrombin fragment 1 + 2, F1 + 2) were performed in platelet poor plasma (PPP) samples. These methods have been shown to be highly sensitve and to allow a close to the in‐vivo situation assessment of how various impacts affect the coagulation status (Waha et al., 2015). Endothelial activation was assessed by measuring plasma levels of tissue factor (TF). Whereby, the portion of TF derived from monocytes was not considered. Treatment‐induced plasma volume changes were derived from the respective hematocrit changes.

Fasting blood samples were taken before and immediately after the treatment from a vein in the antecubital fossa with a 17‐G, 40 mm Teflon® cannula and collected into pre‐citrated Vacuette® marked tubes (Greiner Bio‐one GmbH, Kremsmünster, Austria), containing 500 μL of 3.8% sodium citrate. The citrated blood samples were analyzed within 3 h after collection (TEM, hematocrit, platelet counts). An aliquot was centrifuged at room temperature for 15 min at 1200 *g* to obtain PPP for subsequent determination of thrombin generation, endothelial activation and pro‐ and anticoagulant proteins. Thus, plasma samples were once‐frozen and thawed.

### 2.4 Blood cell counts

Hematocrit and platelet counts were determined on a Sysmex KX‐21 N Automated Hematology Analyzer from Sysmex, Illinois, USA.

### 2.5 Tissue factor triggered TEM assay

The TEM coagulation analyzer (ROTEM®05) was purchased from Matel Medizintechnik, Graz, Austria. Using the thrombelastometer, we obtained the following values: Coagulation Time (CT), the period of time from initiation of the test to the initial fibrin formation; Clot Formation Time (CFT), time of beginning of clot formation until the amplitude of thrombelastogram reaches 20 mm; Maximum Clot Firmness (MCF), expressing the maximum strength in millimeters of the final clot; and alpha, the angle between the line in the middle of the TEM tracing and the line tangential to the developing “body” of the TEM tracing. The alpha angle represents the acceleration (kinetics) of fibrin build up and cross‐linking. The final sample volume was 340 μL. Clot formation was initiated by addition of 40 μL of “trigger solution” containing TF and CaCl_2_ (0.35 pm and 3 mM final concentration, respectively) to 300 μL of citrated blood. This method has recently been described in detail by Sorensen et al. (Sorensen et al., 2003).

### 2.6 Automated fluorogenic measurement of the thrombin generation

Thrombin generation curves were monitored by means of calibrated automated thrombography (CAT) purchased from Thrombinoscope BV, Maastricht, the Netherlands, as described previously (Hemker et al., 2003). The ability of a given plasma sample to generate thrombin was assessed with respect to lag time preceding the thrombin burst (Lag Time, LT), endogenous thrombin potential (ETP), peak height (Peak), time to peak (ttPeak), peak rate of thrombin generation [peak thrombin/(peak time—lag time)] (VELINDEX), and the time point at which free thrombin has disappeared (StartTail). Measurements were carried out in the presence of low amounts (5 pmol/L final concentration) of tissue factor (TF), which allows sensitive detection of thrombin formation.

### 2.7 Standard coagulation tests

Plasma levels of F 1 + 2 were determined by means of ELISA kits from Behring Diagnostics GmbH, Marburg, Germany. Tissue factor (TF) was determined by means of the assay ACTICHROME Tissue Factor ELISA from American Diagnostica, Pfungstadt, Germany. Determinations of plasma levels of FII, FVII, FVIII, and protein C (PC) were performed using a Atellica COAG 360 Coagulation system (Siemens Healthcare Diagnostics GmbH, Vienna, Austria). Tissue‐Plasminogen Activator (tPA) concentrations were determined by means of the IMUBIND t‐PA ELISA kit from American Diagnostica, Pfungstadt, Germany.

## 3 STATISTICS

**FIGURE 1:**
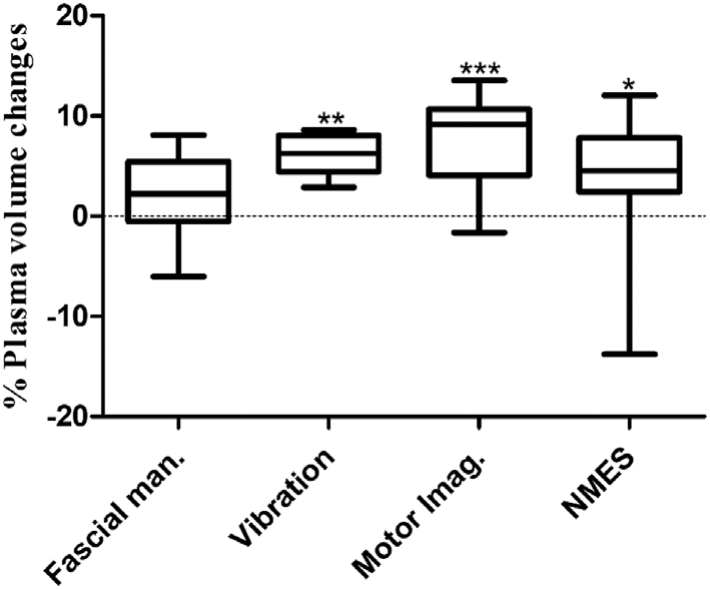
Plasma volume changes due to fascial manipulation, vibration exercise, motor imagery, or neuro‐muscular electrical stimulation. Application of VE, MI, or NMES caused significant increases in plasma volumes. *p*‐values were calculated by means of Kruskal–Wallis test with Dunn's multiple comparison test. *p < 0.05, **p < 0.01, ***p < 0.0001.

Statistical analysis was performed using GraphPad Prism software version 8. Data are presented as means ± SD. The Kolmogorow–Smirnov test was applied to investigate if the variables were normally distributed. Differences between pre and posttreatment samples were determined by means of the Wilcoxon matched‐pairs signed rank test (Tables 1, 2, 3, 4). *p*‐values of plasma volume changes were calculated by means of the Kruskal‐Wallis test with Dunn's multiple comparison test (Figure 1). Statistical significance was set at *p* < 0.05. * *p* ≤ 0.05, ** *p* ≤ 0.01, *** *p* ≤ 0.001.

**TABLE 1:** Effects of fascial manipulation on various coagulation values in 10 young individuals.

	Pretreatment	Posttreatment	*p*‐value
Thrombelastometry (TEM)			
Coagulation time (CT, s)	213 ± 99	243 ± 162	0.3586
Clot formation time (CFT, s)	191 ± 108	176 ± 126	0.2324
Maximum clot firmness (MCF, mm)	51.4 ± 7.9	53.8 ± 8.9	0.0982
Alpha angle (°)	57.9 ± 12.8	60.6 ± 13.8	0.1846
Calibrated automated thrombogram (CAT)			
Lag time (LT, min)	3.41 ± 1.30	3.51 ± 1.49	0.7995
Endogenous thrombin potential (ETP, nM·min)	1739 ± 553	1742 ± 509	0.8457
Peak (nM)	348 ± 115	341 ± 114	1.0000
Time to peak (ttPeak, min)	6.93 ± 1.90	7.22 ± 2.30	0.4258
VELINDEX (nM/min)	112 ± 72	104 ± 62	0.4316
StartTail (min)	22.0 ± 2.4	22.5 ± 2.8	0.1563
Thrombin generation			
Prothrombin fragment 1 + 2 (F1 + 2, pM)	647 ± 333	552 ± 314	0.3750
Endothelial activation			
Tissue factor (TF, pg/mL)	9.76 ± 4.50	8.27 ± 3.06	0.1055
Hematocrit (Hct, %)	40.8 ± 4.2	40.3 ± 4.1	0.1641
Platelet count (10^3^/mL)	222 ± 31	209 ± 51	0.0742

*Note*: Data are expressed as mean ± SD. *p*‐values were calculated by means of the Wilcoxon matched‐pairs signed rank test.

## 4 RESULTS

### 4.1 Effects of fascial manipulation on the coagulation system

No significant effects of FM on the coagulation system were observed. TEM, CAT, endothelial activation, and blood cell parameters were virtually the same in pre and posttreatment samples, listed in Table 1.

### 4.2 Effects of vibration exercise on the coagulation system

VE caused a significant coagulation activation, listed in Table 2. TEM‐derived CTs and CFTs were significantly shortened and MCFs and alpha angles were significantly increased after treatment indicating enhanced clot formation in the posttreatment compared with pretreatment samples. Correspondingly, thrombin generation values (CAT, F1 + 2) were elevated after treatment, indicating a hypercoagulable state of the posttreatment samples. TF levels were not affected by VE, but hematocrit levels were significantly lower after treatment.

**TABLE 2:** Effects of vibration on various coagulation values in 10 young individuals.

	Pretreatment	Posttreatment	*p*‐value
Thrombelastometry (TEM)			
Coagulation time (CT, s)	209 ± 34	187 ± 41	0.0098
Clot formation time (CFT, s)	151 ± 43	127 ± 34	0.0488
Maximum clot firmness (MCF, mm)	55.9 ± 6.2	59.5 ± 4.8	0.0092
Alpha angle (°)	59.9 ± 7.1	66.6 ± 5.5	0.0091
Calibrated automated thrombogram (CAT)			
Lag time (LT, min)	4.09 ± 1.14	3.73 ± 0.88	0.0138
Endogenous thrombin potential (ETP, nM·min)	1354 ± 300	1404 ± 296	0.0488
Peak (nM)	235 ± 88	268 ± 82	0.0020
Time to peak (ttPeak, min)	8.37 ± 1.96	7.32 ± 1.76	0.0091
VELINDEX (nM/min)	64 ± 44	85 ± 53	0.0098
StartTail (min)	23.0 ± 2.6	22.0 ± 2.1	0.0144
Thrombin generation			
Prothrombin fragment 1 + 2 (F1 + 2, pM)	315 ± 161	496 ± 270	0.0488
Endothelial activation			
Tissue factor (TF, pg/mL)	6.43 ± 2.33	7.29 ± 2.51	0.2324
Hematocrit (Hct, %)	39.9 ± 3.9	38.5 ± 3.9	0.0020
Platelet count (10^3^/mL)	234 ± 57	209 ± 55	0.0020

*Note*: Data are expressed as mean ± SD. *p*‐values were calculated by means of the Wilcoxon matched‐pairs signed rank test.

### 4.3 Effects of vibration on FII, FVII, FVIII, PC, and t‐PA plasma levels

No significant effects of vibration were observed with respect to plasma levels (pre vs. posttreatment) of FII (133 ± 9 vs. 132 ± 10, *p* = 0.5271), FVII (114 ± 26 vs. 114 ± 22, *p* = 0.9438), FVIII (137 ± 52 vs. 134 ± 49, *p* = 0.8783) Protein C (116 ± 24 vs. 116 ± 20, *p* = 0.9183), and tissue‐plasminogen activator (4.78 ± 2.88 vs. 4.57 ± 3.03, *p* = 0.0969).

### 4.4 Effects of motor imagery on the coagulation system

Virtually no effects of MI on the coagulation system was observed, listed in Table 3. Solely CAT‐derived LTs were slighty but significantly shortened, indicating an earlier onset of thrombin formation in the posttreatment compared with pretreatment samples. As already shown for VE, hematocrit levels were significantly lower after MI.

**TABLE 3:** Effects of motor imagery on various coagulation values in 10 young individuals.

	Pretreatment	Posttreatment	*p*‐value
Thrombelastometry (TEM)			
Coagulation time (CT, s)	209 ± 47	200 ± 55	0.2220
Clot formation time (CFT, s)	143 ± 38	132 ± 30	0.2020
Maximum clot firmness (MCF, mm)	56.7 ± 3.4	56.6 ± 3.7	0.7165
Alpha angle (°)	63.2 ± 5.2	64.5 ± 5.3	0.3571
Calibrated automated thrombogram (CAT)			
Lag time (LT, min)	4.07 ± 1.14	3.88 ± 1.16	0.0494
Endogenous thrombin potential (ETP, nM·min)	1364 ± 412	1423 ± 574	0.5566
Peak (nM)	246 ± 90	260 ± 103	0.2324
Time to peak (ttPeak, min)	8.06 ± 1.94	7.65 ± 2.13	0.1055
VELINDEX (nM/min)	71 ± 51	81 ± 60	0.1055
StartTail (min)	22.4 ± 2.4	22.4 ± 2.8	0.9219
Thrombin generation			
Prothrombin fragment 1 + 2 (F1 + 2, pM)	367 ± 137	441 ± 210	0.4258
Endothelial activation			
Tissue factor (TF, pg/mL)	6.31 ± 2.75	7.54 ± 4.08	0.2324
Hematocrit (Hct, %)	41.1 ± 3.1	39.4 ± 3.0	0.0039
Platelet count (10^3^/mL)	199 ± 39	164 ± 46	0.0039

*Note*: Data are expressed as mean ± SD. *p*‐values were calculated by means of the Wilcoxon matched‐pairs signed rank test.

### 4.5 Effects of neuro‐muscular electrical stimulation on the coagulation system

Similar to FM, no significant effects of NMES on the coagulation system were observed. TEM, CAT, endothelial activation, and blood cell parameters were virtually the same in pre and posttreatment samples, listed in Table 4.

**TABLE 4:** Effects of NMES on various coagulation values in 10 young individuals.

	Pretreatment	Posttreatment	*p*‐value
Thrombelastometry (TEM)			
Coagulation time (CT, s)	189 ± 31	174 ± 19	0.2210
Clot formation time (CFT, s)	153 ± 63	141 ± 56	0.3008
Maximum clot firmness (MCF, mm)	54.7 ± 7.0	56.1 ± 7.0	0.1807
Alpha angle (°)	62.0 ± 8.2	63.7 ± 8.3	0.3008
Calibrated automated thrombogram (CAT)			
Lag time (LT, min)	4.12 ± 0.96	3.95 ± 0.72	0.4443
Endogenous thrombin potential (ETP, nM·min)	1223 ± 161	1242 ± 194	1.0000
Peak (nM)	233 ± 40	243 ± 42	0.7695
Time to peak (ttPeak, min)	8.11 ± 1.78	7.65 ± 1.13	0.3750
VELINDEX (nM/min)	65 ± 29	68 ± 19	0.6250
StartTail (min)	22.3 ± 2.7	21.7 ± 1.6	0.3583
Thrombin generation			
Prothrombin fragment 1 + 2 (F1 + 2, pM)	421 ± 239	356 ± 109	0.5726
Endothelial activation			
Tissue factor (TF, pg/mL)	9.47 ± 4.88	8.06 ± 2.55	0.6250
Hematocrit (Hct, %)	40.8 ± 3.2	39.9 ± 2.6	0.0827
Platelet count (10^3^/mL)	211 ± 52	207 ± 44	0.3586

*Note*: Data are expressed as mean ± SD. *p*‐values were calculated by means of the Wilcoxon matched‐pairs signed rank test.

### 4.6 Effects of fascial manipulation, vibration exercise, motor imagery, and neuro‐muscular electrical stimulation on plasma volume changes

Percent changes in plasma volume were derived from pre and posttreatment hematocrit values according to Beaumont et al. (Van Beaumont, 1972). FM had no effect on plasma volume, but VE, MI, and NMES caused significant increased plasma volumes, shown in Figure 1.

## 5 DISCUSSION

Numerous studies have reported on the effects of various forms of exercise/intervention on the haemostatic system. In general, it is well documented that the extent of coagulation/fibrinolysis activation is related to exercise intensity and duration (El‐Sayed et al., 2004). Moderate exercise intensity has been shown to cause activation of the fibrinolytic system without concomitant coagulation activation, while intense exercise is associated with activation of both coagulation and fibrinolysis. Interestingly, the exercise‐induced coagulation activation has been shown to be significantly higher in patients at a prothrombotic state, for example, in patients with coronary heart disease, than in healthy participants.

The present study deals with the effects of four particular medical/rehabilitation treatments on the haemostatic system: Fascial manipulation, vibration exercise, motor imagery, and neuro‐muscular electrical stimulation. To our knowledge, no studies exist to date dealing with the effects of fascial manipulation, MI, or NMES on the coagulation sytem.

The majority of the studies dealing with the effects of vibration exercise on hemostasis focus on the fibrinolytic system. Vibration‐induced increases of tPA (tissue plasminogen activator) as well as decreases of PAI‐I (plasminogen activator inhibitor I) plasma levels have been reported indicating enhanced fibrinolytic activity due to VE (Boyle & Nagelkirk, 2010; Ghazalian et al., 2014; Nagelkirk et al., 2021; Takashima & Higashi, 1994). Commensurably, Kabata‐Pizuch et al. have shown significantly decreased fibrinogen levels due to vibrotherapy (Kabata‐Pizuch et al., 2021). In our previous study using a setting different to that of the present study, resistance VE did not affect the coagulation system (Waha et al., 2015). In general, data of the effects of VE on the clot formation/dissolution process are scarce to date.

Therefore, we examined in the present study the effects of the four interventions on the haemostatic system, that is, by means of TEM. This technique can objectively reflect the formation, development, and dissolution of the thrombus in WB samples (Liu et al., 2019). We additionally examined the influence of the four interventions not only on several standard coagulation values but also on the capability of the respective plasma samples to generate thrombin by means of CAT. This technique is suitable to detect hyper‐ or hypocoagulability of PPP samples (Hemker et al., 2003).

We found that neither FM, MI, nor NMES affected the coagulation system. Virtually all TEM, CAT, and standard parameters were the same in the pretreatment as in the respective posttreatment samples. We conclude that these three treatments can be applied in the clinical setting or in rehabilitation without inducing activation of the coagulation system and exposing patients to thrombotic events.

In contrast to the three treatments described above, VE exerted significant effects on the coagulation system. All TEM and thrombin generation values (CAT, F1 + 2) indicated an exercise‐induced shift towards hypercoagulability. At first glance, these findings seem to contradict two previous studies reporting no effects of VE on coagulation parameters (Haider et al., 2013; Waha et al., 2015). However, VE in these studies was performed under simulated microgravity (6° head‐down‐tilt) while in the present study VE was performed in an upright position. This requires increased muscle work and causes enhanced hydrostatic pressure in the lower part of the body (Masoud et al., 2008). Moreover, the coagulation measurements in the present study were performed by using low amounts of TF (in the picomolar range) as initiator, a condition allowing very sensitive detection of potential coagulation changes, propably not measurable by using standard coagulation methods (Cvirn, Gallistl, Leschnik, & Muntean, 2003; Cvirn, Gallistl, Rehak, et al., 2003).

Notably, after application of VE, MI, or NMES the plasma volumes were significantly higher in the posttreatment compared with the respective pretreatment samples, indicating a treatment‐induced transmission of fluid from the periphery back to the circulation. This contradicts the findings of various studies showing that most types of acute exercise (cycling, treadmill running, swimming, etc.) are associated with a decrease of plasma volume, and, thus, with haemoconcentration (Goodman et al., 1985; Gore et al., 1992; Maroto‐Sánchez et al., 2019). In these studies, fluid has been shown to be transferred from the blood to the interstitial spaces due to the exercise (El‐Sayed et al., 2004; Kargotich et al., 1998).

Plasma dilution is apparently the primary reason for the decrease of platelet numbers after treatment (−10.7% in VE and −17.6% in MI). Correspondingly, plasma volume changes were 6.3% in VE and 9.2% in MI, respectively. Platelet number were not altered significantly in FM and NMES which were associated with the lowest level of plasma dilution (2.2% and 4.5%, respectively). It cannot be excluded that other mechanisms are at work which should be examined in a future study with a higher number of participants.

An explanation for the treatment‐induced plasma expansion shown in our present study cannot be found in the scientific literature so far. We speculate that the renin angiotensin system (RAS) might be involved in the treatment‐induced plasma expansion. Speretta et al. have shown that mRNA expression of angiotensin converting enzyme 2 (ACE2) is increased after resistance training in an animal model (Speretta et al., 2016). Presumably, increased ACE2 levels trigger the formation angiotensin II which subsequently triggers the secretion of aldosterone in the adrenal cortex. This causes enhanced reabsorption of water in the kidneys with subsequently increased blood volume. Whether this assumption is true for our present study has to be clarified in future research.

It has been shown that at low plasma dilutions (20%–40%), a hypercoagulable state develops, probably due to decreased antithrombin concentrations (Boyd et al., 2021). However, due to the low plasma expansion occurring in our experiments (2%–8%), the effects of the four treatments on the coagulation cascade are likely to be very small.

We suggest that application of MI or NMES brings benefit to patients beyond the standard effects like reducing edema or decreasing atrophy and pain: The blood dilutional effect may be advantageous in delivering oxygen to muscles because of a reduced resistance to blood flow. Additionally, the increase in plasma volume may also contribute to the body water pool and help offset dehydratation (El‐Sayed, 1998). This may be of particular importance for old and/or bedridden patients.

As stated above, a blood dilutional effect was also observed for VE. However, vibration apparently represents such a strong procoagulant stimulus that, despite plasma expansion, postexercise samples were hypercoagulable compared with preexercise samples. We conclude that VE should not be applied to participants vulnerable to thrombotic event, for example, to patients with cardiovascular disorders. It has to be stated, however, that the changes of the coagulation parameters presented herein, although significant, were relatively small and remained within their respective reference ranges (Lamprecht et al., 1985).

The following limitations have to be considered when interpreting the results of our study: Due to the low number of participants, some findings of no observed differences between pre and postexercise samples may represent false negatives. Moreover, only a restricted blood volume was available at each time point, this limited the analyzed number of different parameters of coagulation.

VE is clearly associated with coagulation activation, according to our data, despite its plasma expansion effect. Thus, VE seems to be no suitable medical/rehabilition treatment particularly for patients at a prothrombotic state. Whereby, coagulation changes were relatively small and the number of test subjects is relatively low in our study. For the completely reliable statement, that VE induces marked coagulation activation, a future study with a larger number of test persons is certainly necessary.

It has been shown that the extent of VE‐induced coagulation activation depends on applied duration/frequency/amplitude (El‐Sayed et al., 2004; Ghazalian et al., 2014). Therefore, future studies on VE should determine optimal settings (duration/frequency/amplitude) leading to minimal coagulation activation. This could make VE a suitable medical/rehabilition method for a wide range of subjects/patients.

In conclusion, the findings of the present study suggest that the three treatments FM, MI, and NMES are not accompanied with coagulation activation, and, thus, seem to be suitable to be applied even to patients at an elevated risk for thrombotic events. However, it has been shown that exercise‐induced coagulation activation (due to cycling or running) is higher in patients with a history of thrombotic events than in healthy subjects (El‐Sayed, 2002). Therefore, future studies investigating the effects of FM, MI, and NMES on the coagulation system in participants susceptible for thrombotic events are required.

## AUTHOR CONTRIBUTIONS

Conception and design: Gerhard Cvirn, Nandu Goswami. Acquisition of data for the work: Anna Hawliczek, Bianca Brix, Axel Schlagenhauf, Karin Schmid Zalaudek, Thomas Wagner, Willibald Wonisch, Sebastian Schwaminger Margret Paar, Ziva Arko. All authors approved the final version of the manuscript. All authors agree to be accountable for all aspects of the work in ensuring that questions related to the accuracy or integrity of any part of the work are appropriately investigated and resolved. All persons designated as authors qualify for authorship, and all those who qualify for authorship are listed.

## FUNDING INFORMATION

No funding information provided.

## CONFLICT OF INTEREST STATEMENT

The authors declare no conflict of interests financial or otherwise.

## ETHICS STATEMENT

The study was carried out in accordance with the WMA Declaration of Helsinki. An approval from the Ethics Committee of the Medical University of Graz was obtained before starting the study (EK 30‐268 ex 17/18).

## Data Availability

The data that support the findings of the study are available from the corresponding author upon reasonable request.
